# A Comparison of Dietary Intake and Nutritional Status between Aged Care Residents Consuming Texture-Modified Diets with and without Oral Nutritional Supplements

**DOI:** 10.3390/nu14030669

**Published:** 2022-02-05

**Authors:** Xiaojing Sharon Wu, Lina Yousif, Anna Miles, Andrea Braakhuis

**Affiliations:** 1 Department of Nutrition, Faculty of Medical and Health Sciences, University of Auckland, Auckland 1023, New Zealand; lyou561@aucklanduni.ac.nz (L.Y.); a.braakhuis@auckland.ac.nz (A.B.); 2 Department of Speech Science, School of Psychology, University of Auckland, Auckland 1023, New Zealand; a.miles@auckland.ac.nz

**Keywords:** oral nutritional supplement, malnutrition, dysphagia, texture-modified diet, aged care

## Abstract

Oral nutritional supplements (ONS) are high-energy and protein-rich nutrition drinks that are commonly prescribed to individuals with compromised nutritional status. Aged care residents requiring texture-modified diets are exposed to poor oral intake and malnutrition. This study aimed to investigate the dietary intake and nutritional status of residents consuming texture-modified diets with and without ONS. This multicentre cross-sectional study included 85 residents consuming texture-modified diets (86.0 ± 8.7 y; *n =* 46 requiring ONS and *n =* 39 without ONS). A one-day dietary record was completed using a validated visual plate waste estimation method. To determine the adequacy, nutrition intake was then calculated using FoodWorks (Xyris Ltd., Brisbane, Australia) and compared to the recommended dietary intake for Australia and New Zealand. The Mini-Nutritional Assessment Short Form was collected to assess nutritional status. Residents receiving ONS had significantly higher energy, protein, carbohydrates and fat intake than those who did not consume ONS (*p* < 0.05). No significant differences were found in saturated fat, fibre or sodium intake. With the administration of ONS, residents were able to meet their protein requirement but fell short of their energy and carbohydrates requirements. Both groups had inadequate fibre intake and a high saturated fat intake. A total of 48% of the residents were at risk of malnutrition and 38% were malnourished. Aged care residents requiring texture-modified diets are at high risk of malnutrition as a result of inadequate dietary intake. Administration of ONS may be an effective strategy to optimise nutrition intake.

## 1. Introduction

Texture-modified diets (TMDs) are a common dietary intervention used in aged care facilities for residents who struggle with chewing or swallowing (dysphagia) [1]. The texture and consistency of solid foods and liquid are modified to ease swallowing and prevent choking and aspiration [2]. Based on the International Dysphagia Diet Standardisation Initiative (IDDSI), TMDs are categorised into five levels: level 7—easy to chew/regular; level 6—soft and bite-sized; level 5—minced and moist; level 4—pureed; and level 3—liquidised [3]. When textures are modified, they often result in unappealing, tasteless and nutritionally-dilute food that is not well accepted by consumers [4]. Despite IDDSI providing standardised guidance for healthcare providers with regards to the texture and consistency requirements of each TMD level, the nutrition requirements still need to be addressed.

As the population ages, the prevalence of chewing and swallowing problems becomes more significant in all healthcare settings. Particularly, older adults living in aged care facilities are at a greater risk of dysphagia than those living in the community [5]. Therefore, there is a growing need for TMDs in the last decade, with 30%−47% of residents living in aged care facilities requiring TMDs [6,7,8]. Recent reviews suggest that TMD consumers have significantly lower energy intake and higher risk of malnutrition compared to those having regular diets [9,10]. In addition to increasing morbidity, mortality, hospital length of stay, readmission and costs of care, dysphagia and malnutrition are also consequences of each other [11,12]. A New Zealand study reported that aged care has a significantly higher prevalence of malnutrition and higher risk of dysphagia compared to older adults from hospital or community settings [13]. Several studies have reported that aged care residents consuming TMDs had compromised nutrition intake as a result of poor oral intake and inadequate meal portions [7,14,15]. 

One common approach to solve this problem involves the use of oral nutritional supplements (ONS) [16]. ONS prescription is individualised based on the gap between a resident’s energy/protein requirements and the actual intake obtained from food [17]. It has been proven that ONS are beneficial to older adults who have difficulty maintaining their nutritional intake, in particular, those with compromised nutritional status [18]. Wright et al. reported that ONS were prescribed to 54% of hospital geriatric patients on TMDs [16]. ONS are often high-energy and high-protein drinks that can potentially improve the energy and protein intake, nutritional status and survival of older malnourished adults [19,20]. Dietitians are recommended to prescribe suitable ONS to dysphagic patients at high risk of malnutrition and continually review their compliance, tolerance, weight and nutritional status [21]. Physicians and dietitians are responsible for prescribing ONS to nutritionally vulnerable residents, including inadequate oral intake, unintentional weight loss or poor wound healing [22]. ONS are available in a wide range of formats, brands and flavours which contain various nutritional values. Powder form and ready-mixed liquid form are the most common ONS used in New Zealand [17]. Despite the increased use of ONS, there is a lack of evidence to guide the use of specific ONS types, dosage, time of administration and duration in different patient groups [18,22]. To our knowledge, few studies have examined the impact and compliance of ONS on TMD consumers [23]. In relation to improving dietary intake and nutritional status in aged care residents consuming TMDs, understanding the role of ONS is essential to directing future research. The aims of this study were 1) to compare the dietary intake, adequacy and nutritional status of the residents living in aged care facilities consuming TMDs with and without ONS and 2) to examine residents’ compliance with ONS.

## 2. Materials and Methods

This was a cross-sectional study conducted in five Auckland aged care facilities as part of the post-IDDSI implementation data collection in a larger project (under peer review). The STROBE statement for observational studies was followed in the design of this study [24]. The project evaluated and facilitated the implementation of IDDSI in participating aged care facilities in order to increase TMD compliance and staff understanding. In this study, a quantitative approach was employed to investigate the role of ONS and provide extensive information on the nutritional perspectives of residents consuming TMDs. Data on individuals was collected in a single day over 26 days between June 2020 and March 2021. Three facilities had to delay the data collection due to COVID-19 lockdowns. The study was approved by The University of Auckland Human Participants Ethics Committee (023048) on 28 June 2019.

### 2.1. Subjects

Residents over 65 years old who were on TMD without enteral nutrition support and residing in one of the participating aged care facilities were eligible to participate in the study. Residents who were receiving palliative care, respite care or did not consume full TMD routinely were excluded. The five aged care facilities were recruited through convenience sampling with the strategy to include a range of small and large facilities. Facilities provided IDDSI-compliant TMDs, including soft and bite-sized, minced and moist, and pureed levels. 

### 2.2. Data Collection

Data collection was completed by a researcher (student dietitian) (L.Y). A standard data extraction form was used to collect resident demographic information from their aged care clinical record, including age, gender, ethnicity, height, length of stay, mobility level, medical history, the reason for requiring TMDs, level of TMDs and thickened fluids. Residents were weighed at least monthly, thus the most recent weight and the weights from the previous three months were collected from their clinical record. Body mass index (BMI) was then calculated using height and weight records. 

#### 2.2.1. Dietary Assessment

A plate wastage sheet was completed for individual residents where all foods and beverages were weighed and recorded over 24 hours, including ONS. A maximum of five residents were observed in a day. The researcher was present in the facility before breakfast was served and stayed until supper. The night shift staff recorded any food or drink consumed by the resident outside of this period. The approach adopted a previously validated visual plate wastage estimation method that is applicable to different meal textures and varieties and has been used in aged care facilities [25,26]. Before serving, each food/drink item was weighed twice to the nearest gram (Salter Electronic Disk scales, Kent, UK) and recorded as a standard serving size. After residents had finished their food, the researcher used visual estimation to record the amount of food/drink/ONS that was consumed (‘all left (100%)’, ‘mouthful eaten (90%)’, ‘3/4 left (75%)’, ‘1/2 left (50%)’, ‘1/4 left (25%)’, ‘mouthful left (10%)’ and ‘none left (0%)’). The amount of each food item consumed was then calculated by subtracting the amount of waste portion from the weighed standard serving size in the sample item. Each food/drink item was also photographed before and after consumption. To minimise rater bias, another dietitian researcher (X.W) used the photos to estimate the plate wastage independently. Where disagreement arose, a third author (A.B) was consulted to meet consensus. 

#### 2.2.2. Dietary Requirement

The one-day dietary intake was entered and analysed using FoodWorks, version 10 (Xyris Ltd., Brisbane, Australia). This study analysed all available nutrients, including macronutrients: energy, protein, carbohydrates, fat, saturated fat, fibre and sodium. Other micronutrients were unavailable since the nutrition information was not listed for pre-packaged foods. To assess nutritional adequacy, dietary intakes were then compared with the recommended dietary intake (RDI) for Australian and New Zealand adults aged over 70 years old [27]. The estimated energy requirement (EER) was calculated using individual resident age, gender, BMI and physical activity level. Protein requirement was estimated using resident weight (1.07 g/kg for men and 0.94 g/kg for women). Carbohydrates and fat intake were recommended in relation to their contribution to total dietary energy. Adequate intake value was used for fibre and sodium.

#### 2.2.3. Nutritional Status

The Mini Nutrition Assessment-Short Form (MNA-SF) is one of the most commonly used nutrition screening tools in aged care settings covering oral intake, weight loss, BMI, mobility, psychological and acute conditions [28,29]. Where residents had cognitive impairment, the researcher administrated the MNA-SF based on information obtained from resident clinical records and interviewing resident key workers. A score of less than 8 was identified as malnourished; 8–11 was recognised as at risk of malnutrition, and 12–14 was considered as normal nutritional status [30].

### 2.3. Data Analysis

Statistical analyses were performed using GraphPad Prism (v9.0; GraphPad Software, Inc., San Diego, CA, USA). A descriptive analysis of all explanatory variables was conducted and presented as mean and standard deviation for normal distribution. Median and interquartile range were reported for non-normal distribution. Compliance with the ONS was calculated for each resident based on the percentage of actual ONS consumed. To determine whether raw data distributions within each group were normal, we performed the Shapiro–Wilk test (normal distributions are defined as *p* > 0.05). In order to compare the nutrition intake and nutritional status between TMDs with and without ONS consumption, Welch’s *t*-test was used for normally distributed parameters and the Mann–Whitney test was used for non-normally distributed parameters. To explore the associations of ONS consumption with resident characteristics, Fisher’s exact test or Chi-square was performed comparing the group that consumed ONS with TMDs and the group that only had TMDs. Linear regression was used to investigate predictors of total energy intake and nutritional status (MNA-SF score). Statistical significance was determined by a *p*-value less than 0.05.

## 3. Results

Among the 425 residents living in the facilities, 20% (*n =* 85) who required full TMDs were considered eligible. Of these, 46 residents required routine ONS administration. Resident characteristics are summarised in Table 1. No significance was found in demographic distribution between the ONS consumption group and the non-ONS consumption group. There was a significant correlation between the level of TMD prescribed and ONS consumption. Among those who consumed a minced and moist diet, only one resident did not require ONS. Three medical conditions were also found to be significantly associated with the use of ONS, including diabetes, stroke and thyroid dysfunction. 

### 3.1. Dietary Intake

The average daily energy, protein, carbohydrates and fat intake were significantly higher among residents who consumed TMDs with ONS compared with those who did not have ONS (Table 2). There were no differences in saturated fat, fibre and sodium intake between the two groups. When comparing the daily nutrition intake to the RDI, only fat intake was within the recommended range without ONS. Protein intake was adequate when residents were given ONS.

Fortsip powder was the most common type of ONS and was used in all facilities (*n =* 34), followed by Ensure (*n =* 8) with 32% (*n =* 15) of the residents receiving ready-to-drink bottles. ONS powder was mixed with water or made into a milkshake. Other uncommon ONS including Beneprotein, Diasip, Cubitan, Advital, Impact advanced recovery and Calogen were administrated to one or two participants based on their medical conditions. Three times a day was the most common frequency (46%, *n =* 21), followed by four to five times a day (28%, *n =* 13) and twice a day (22%, *n =* 10). The average daily compliance to ONS was 86%, with a range from 17% to 100%. More than half of the residents were able to drink all the ONS provided (63%, *n =* 29). Among all residents, 15% (*n =* 7) were able to finish ≥75% of the ONS and 11% (*n =* 5) consumed 50–67% of prescribed ONS. Nine percent (*n =* 7) of the residents had a compliance rate below 50%.

ONS contributed 35% energy (558 ± 256 kcal), 38% protein (26 ± 10 g), 36% carbohydrate (68 ± 33 g) and 28% (18 ± 17 g) fat intake to the overall average daily intake. Without ONS, the two groups had no differences in energy (1011 ± 355 vs. 1169 ± 413 kcal, *p* = 0.08), protein (43 ± 19 vs. 49 ± 16 g, *p* = 0.1), carbohydrate (119 ± 46 vs. 126 ± 42 g, *p* = 0.48) and fat (41 ± 16 vs. 50 ± 29 g, *p* = 0.21) intake. As shown in Table 3, a higher proportion of residents who consumed TMDs with ONS were able to meet their macronutrient requirements as compared to residents who did not have ONS.

### 3.2. Nutritional Status

Table 4 compares the nutritional status and MNA-SF measurements between the ONS consumption group and the non-ONS consumption group. Residents who required ONS had significantly lower weight and BMI. They also scored lower in MNA-SF. When compared to individuals who did not require ONS, the proportion of well-nourished residents in the ONS consumption group was significantly lower. In total, 51% (*n =* 43) and 32% (*n =* 27) of the residents had neuropsychological problems. A positive association was only found between the consumption of ONS and energy intake as indicated by the multiple linear regression analysis (Table 5). The MNA-SF model identified four contributors, including age, feeding assistance, TMD level and BMI. The negative association of age and feeding assistance indicated that both older residents and residents requiring more feeding assistance scored lower in MNA-SF. In contrast, the less food modification required (higher level of TMDs) and the greater the BMI, the higher MNA-SF was scored.

## 4. Discussion

To our knowledge, this is the first study assessing the nutrition adequacy and evaluating the role of ONS in New Zealand aged care residents consuming TMDs. For residents who consumed ONS, their ONS was a notable contribution to their daily energy and protein intake. ONS consumers had significantly higher nutrition intake but lower MNA-SF scores and weights, and there was a lower percentage of well-nourished individuals (MNA-SF ≥ 12). Findings suggest that the prescription of ONS was associated with several resident characteristics, including the TMD level, medical conditions and nutritional status. These results support the recommendation by Holdoway and Smith for dysphagic patients at high risk of malnutrition to receive ONS [12]. Previous studies revealed that the level of TMDs patients received is related to medical conditions such as dementia [31]. Although previous studies indicate that the reasons behind TMD orders are unclear, our study concurs with Wright’s and Miles et al.’s findings that medical status and poor dental health were significant factors in the TMD prescription [7,16,32]. In contrast to Canadian and Australian aged-care facilities, both Miles and our study found that Level 4 puree diets were the most common type of TMDs rather than Level 6 soft and bite-sized or Level 5 minced and moist diets [7,33,34]. The small number of residents consuming minced and moist diets limits the ability to make conclusions in ONS prescription among TMD levels. Moreover, we found no significant correlation between demographic and energy intake based on a multiple linear regression analysis, which suggests the study population was relatively homogeneous. Future research should include larger groups of participants from each level of TMD in order to determine whether ONS use has a direct association with TMD levels.

### 4.1. Nutrition Intake and Adequacy

Studies that compared the nutritional intake of TMDs and regular diets have established that aged care TMD consumers have insufficient energy and protein intake [14,15,26,35]. The average energy and protein intake results from our study were similar to the results from other studies assessing aged care TMD consumers. Without any nutritional intervention, the average energy and protein intake ranged between 908 kcal to 1764 kcal and 42 g to 70 g, respectively [14,26]. The variance in TMD levels can explain the noticeable differences in nutrition intake. Puree diets are usually less appealing and more nutrient-diluted due to the higher level of texture and consistency modification [4,36]. The significant contribution of ONS to macronutrients is noticeable; similar effectiveness was found by Wright et al. in hospitalised geriatric patients consuming TMDs [16]. The positive association between ONS consumption and energy intake is also supported by a recent review which concluded that improved macronutrient intake can be achieved by providing ONS [9]. Our results demonstrate that ONS intake does not reduce the amount of food consumed. Therefore, ONS can be an effective addition to residents’ usual diets giving the benefits of increased macronutrient intake. With the appropriate use of ONS, residents were more likely to meet their nutritional requirements. Although some studies suggested ONS consumption may lead to decreased appetite and therefore reduced nutrition intake from the food, it needs to be further examined in TMD consumers [22,37]. The volume and frequency of ONS are required to be monitored and adjusted as nutritional status improves [38]. Despite only protein and fat achieving recommended daily intake, a previous ONS and food trial demonstrated improvements in albumin, total iron-binding capacity, transferrin and body weight when the nutritional intake of residents reached 100% of recommended values for key macro and micronutrients [23]. The inadequate dietary intake can be a result of loss of appetite, inadequate nutrient provision from the menu, poor meal compliance, impaired chewing and swallowing function and reduced motivation to eat due to social isolation or depression [7,16,26,39]. A significant amount of research has demonstrated that aged care menus are significantly low in nutrients and below the RDI, in particular, pureed menus [26,35,36,40,41,42]. The compromised carbohydrate intake can be explained by the similar results found in New Zealand aged care facilities where only 26%, 63% and 79% of the TMD menus offered adequate portion sizes of carbohydrate, protein and vegetables, respectively [43]. To ensure the nutrition quality of TMD menus, dietitians should conduct menu audits regularly [44]. Foodservice staff are required to follow dietitian recommended standardised recipes to allow consistent and highly nutritious meal productions [40]. 

Despite the positive impact of ONS on macronutrients, the fibre intake was concerningly low and almost identical between the two observation groups. The inadequate fibre intake is consistent with a previous review reporting low fibre consumption in all studies investigating TMDs [9]. Fibrous content can be destroyed during food modification which leads to lower fibre components in TMD menus compared to regular menus [36]. Consumption of insufficient fibre is associated with a greater risk of constipation, particularly among aged care residents with high prevalence of constipation [45]. Residents on pureed diets receiving fibre-fortified cereal along with ONS reported a low fibre intake of 12 g per day [23]. Although there was limited evidence investigating fibre enrichment in TMD consumers, Cruz-Jentoft et al. found a compelling result using protein and fibre enriched ONS (20% protein, 2 g fibre/100 mL) in residents who are undernourished or at risk of malnutrition, which improved both nutritional status and bowel movement and reduced digestive symptoms [46]. Further studies are required to explore the effect on improved fibre intake in TMD consumers. 

### 4.2. ONS Compliance

Although there has been an increased prescription of ONS in aged care facilities, the prescription standards of ONS are still inconclusive [18]. A 54% prevalence of ONS administration in our study is similar to Vucea et al.’s report showing that 57% of puree diet residents were on ONS [47]. The compliance of ONS is a significant contributor to the effectiveness of ONS on nutrition intake and nutritional status. Although long-term compliance is arguable, Hubbard et al.’s review found no significant relationship between ONS compliance and the duration of ONS consumption or the ONS variety [48]. ONS compliance in TMD consumers has been less investigated. Our study found the TMD consumers had 8% higher compliance than the 78% overall mean compliance extracted from 46 studies of ONS [48]. This result agrees with the findings of other studies investigating ONS compliance in residents who are undernourished or at risk of malnutrition, in which ONS was well accepted for three to six months [23,46]. Aged care residents are more likely to have better compliance compared to hospital patients. The fact that ONS is routinely served by familiar nurses and healthcare assistants could encourage consumption. Front line clinical staff awareness and assistance can effectively improve resident nutrition intake and prevent weight loss, including supplement administration [49]. Although ONS were commonly served at mid-meals, it was hard to analyse the timing of ONS administration due to the large heterogeneities among facilities and resident needs. According to de Sa and colleagues, ONS is more accepted at afternoon tea and morning tea, and it was better complied by TMD patients than those on regular diets [50]. Besides ONS, other sources of nutrition support should also be considered to use in combination with ONS, such as dietitian counselling and food-first strategy [17,22,51]. By nutrient fortification and/or providing small and frequent meals, the food-first strategy had positive effects on nutritionally vulnerable older adults [9]. 

### 4.3. Nutritional Status

It is evident that inadequate nutrition intake from consuming TMDs can have a detrimental effect on both nutritional and functional status [52]. Residents with (94%) or without ONS (77%) consumption both had a high prevalence of malnutrition or at risk of malnutrition. These results were consistent with previous local aged care investigations [13,53]. The high prevalence of malnutrition implies the importance of assessing the risk of malnutrition in those with dysphagia due to their close associations [54]. The current study supports the existing literature which provides evidence for the importance of malnutrition screening and continual assessment in the aged care setting in order to reduce the risk of complications [11]. Besides the common factors contributing to compromised nutritional status, such as weight, BMI and age, our study proved that increased demand for feeding assistance and advanced modifications in TMDs were also important factors in predicting nutritional status. Although the average MNA-SF score of 8.3 (2.9) indicates a risk of malnutrition, some residents had reasonable meal consumption and/or insignificant weight loss. The low score was limited to the high prevalence of dementia/psychological disorders (83%) and immobility (69%). Vucea et al. confirmed this finding in a larger cross-sectional study, with an 8.5 (2.4) average MNA-SF score among pureed diet indicating residents with diagnosed dementia and high level of cognitive impairment had significantly lower MNA-SF scores [47]. The prescription of ONS was most likely related to the higher prevalence of compromised nutritional status (94%), as evidenced by the lower weight, BMI and MNA-SF score in the ONS consumption group. This finding matches the observation in Miles et al.’s study, which demonstrated the significant association between decreased weight or BMI and increased number of weeks on nutrient-fortified TMDs [55]. Despite few studies examining the impact of ONS on nutritional status change in TMD consumers, Welch et al. demonstrated significant weight improvement after three months and six months of ONS consumption. A review concluded that older adults would have a small but consistent weight gain with the addition of ONS, while their mortality risk would also be significantly reduced if they were malnourished prior to commencement of ONS [56]. Improved MNA-SF score, weight, BMI and functional status were found in a three-month ONS intervention in Spanish aged care residents who had been diagnosed with malnutrition [57].

### 4.4. Limitations and Future Directions

The results from this study are consistent with the findings obtained in other international aged care setting studies and will inform future intervention development. All facilities had implemented IDDSI, therefore, the terminologies and descriptions were consistent. However, this research is subject to certain limitations due to the cross-sectional design. Without longitudinal data, it was impossible to determine the long-term causal relationship between ONS consumption and nutrition intake or nutritional status. Although we included all eligible participants from each facility, facilities were selected through convenience sampling. Volunteer facilities may have better foodservice practices, and therefore the nutrition intake and nutritional status may be overestimated. While plate wastage estimation is a validated measure of food consumption, we only collected a one-day food record which may not be representable to their usual overall dietary patterns.

Further, mealtime assistance and dining environment also play important roles in optimising resident oral intake [58,59]. Unfortunately, these factors were not analysed in this study. This was not a randomised clinical trial with a randomised allocation of matched residents into ONS and non-ONS and instead offers an interesting observation of differences between those with dysphagia prescribed ONS and those who are not. 

Although ONS can be an effective intervention to improve dietary intake and nutritional status, the long-term cost needs to be considered. Food fortification may be an alternative cost-effective way to improve intake by having more nutrient-dense TMDs [9]. To avoid unnecessary prescribing and inappropriate use, dietitians should be consulted for initial assessment and continual review. Additionally, when choosing the type of ONS, dietitians should consider the thickening level of the drink in order to avoid risks associated with viscosity [60]. 

## 5. Conclusions

Aged care residents on TMDs are at higher risk of inadequate nutrition intake and malnutrition. To optimise oral intake, it may be necessary to consider offering nutrient-dense food or drinks, such as ONS. An individual dietitian assessment should be provided to at-risk residents. Dietitians should also collaborate with foodservice staff to create standardised TMD menus that provide sufficient nutrients to ensure residents are able to consume adequate nutritional intake. Although prescription of ONS provides promising results in improving resident intake, long-term compliance and nutritional status need to be monitored. Our study has demonstrated that ONS is well-accepted by aged care TMD consumers, and consumption of ONS was associated with increased daily intake. Further research is required to continue to explore whether ONS consumption is positively associated with nutrition intake and nutritional status in TMD consumers.

## Figures and Tables

**Table 1:** Residents’ characteristics associated with and without oral nutritional supplements.

Resident Characteristics	Total	TMDs with ONS	TMDs without ONS	*p*-Value
Number of residents % (*n*)	100% (85)	54% (46)	46% (39)	
Age (years), median (IQR)	88 (81, 91)	88 (80, 90)	89 (83, 93)	0.27
Gender % (*n*)				0.11
Female	68% (58)	76% (35)	59% (23)	
Male	32% (27)	24% (11)	41% (16)	
Ethnicity % (*n*)				*x*^2^ (4) = 4.25 *p* = 0.37
NZ European	48% (41)	43% (20)	54% (21)	
Other European	33% (28)	39% (18)	26% (10)	
Asian	8% (7)	11% (5)	5% (2)	
Pacific and Maori	6% (5)	4% (2)	8% (3)	
Indian and Middle Eastern	5% (4)	2% (1)	8% (3)	
Level of Care % (*n*)				*x*^2^ (2) = 0.94, *p* = 0.62
Hospital	82% (70)	80% (37)	85% (33)	
Rest home	17% (14)	17% (8)	15% (6)	
Dementia	1% (1)	2% (1)	-	
Average length of stay (months), median (IQR)	35.0 (18.5, 520)	38.5 (19.8, 58.5)	34.0 (17.0, 50.0)	0.39
Mobility level % (*n*)				*x*^2^ (2) = 0.84, *p* = 0.66
Mobile	26% (22)	24% (11)	28% (11)	
Semi-mobile	31% (26)	35% (16)	26% (10)	
Immobile	44% (37)	41% (19)	46% (18)	
Medical Condition % (*n*)				*x*^2^ (13) = 18.0, *p* = 0.16
Arthritis	22% (19)	26% (12)	18% (7)	0.44
Brain injury	6% (5)	4% (2)	8% (3)	0.66
Cardiovascular conditions	39% (33)	37% (17)	41% (16)	0.82
Diabetes *	15% (13)	11% (5)	21% (8)	0.002
Dementia	60% (51)	61% (28)	59% (23)	>0.99
Depression or anxiety	21% (18)	20% (9)	23% (9)	0.79
Hypertension	44% (37)	39% (18)	49% (19)	0.39
Huntington’s Disease	1% (1)	2% (1)	0% (0)	>0.99
Gastrointestinal conditions	19% (16)	11% (5)	28% (11)	0.05
Osteoporosis	15% (13)	17% (8)	13% (5)	0.76
Pulmonary conditions	9% (8)	13% (6)	5% (2)	0.28
Parkinson’s Disease	5% (4)	7% (3)	3% (1)	0.62
Stroke *	8% (7)	2% (1)	15% (6)	0.04
Thyroid Dysfunction *	21% (18)	30% (14)	10% (4)	0.03
Reason requiring TMD % (*n*)				*x*^2^ (3) = 1.64, *p* = 0.65
Swallowing difficulties	44% (37)	41% (19)	46% (18)	
Chewing difficulties	27% (23)	24% (11)	31% (12)	
Dementia	27% (23)	33% (15)	21% (8)	
Others (vision or tolerance)	2% (2)	2% (1)	3% (1)	
Level of TMDs prescribed % (*n*) *				*x*^2^ (2) = 6.89, *p* = 0.03
Soft and bite-sized	41% (35)	37% (17)	46% (18)	
Minced and Moist	13% (11)	22% (10)	2% (1)	
Pureed	46% (39)	41% (19)	43% (20)	
Thin Fluids % (*n*)	74% (63)	78% (36)	69% (27)	0.46
Thickened Fluids % (*n*)				*x*^2^ (2) = 3.27, *p* = 0.19
Extremely thickened	1% (1)	0% (0)	3% (1)	
Moderately thickened	8% (7)	11% (5)	5% (2)	
Mildly thickened	16% (14)	11% (5)	23% (9)	
Feeding assistance % (*n*)				*x*^2^ (2) = 1.70, *p* = 0.43
Full Assistance	51% (43)	57% (26)	44% (17)	
Partial Assistance (set-up)	11% (9)	11% (5)	10% (4)	
Independent	39% (33)	33% (15)	46% (18)	

Note: ONS = Oral Nutritional Supplement; TMD = Texture-Modified Diet; Chi-square and Fisher’s Exact tests were conducted to test the significance between the two groups. * *p* < 0.05 is considered significant.

**Table 2:** Average (SD) daily nutrition intake and requirement of residents consuming texture-modified diets with and without oral nutritional supplements.

	^^^ RDI (>70 yr)	All TMDs *n =* 85	TMDs with ONS *n =* 46	TMDs without ONS *n =* 39	*p*-Value
Energy (kcal)	1620 (229)	1378 (473)	1554 (1324, 1821)	1155 (869, 1329)	<0.0001
Protein (g)	55 (44, 65)	59 (44, 72)	63 (53, 81)	46 (38, 63)	<0.0001
Carbohydrates (g)	182–263	154 (57)	179 (57)	126 (42)	<0.0001
Fat (g)	36–63	50 (39, 63)	55 (44, 65)	41 (32, 61)	0.02
Saturated fat (g)	14–18	22 (15, 28)	23 (17, 29)	20 (13, 28)	0.43
Fibre (g)	25 (Female) 30 (Male) *	13 (11, 17)	13 (10, 17)	12 (11, 17)	0.94
Sodium (mg)	2000 *	1560 (606)	1640 (667)	1467 (519)	0.18

Note: Normally distributed variables are presented as mean (standard deviation) and non-normally distributed variables are presented as median (interquartile range 25%, 75%). RDI = Recommended Dietary Intake (National Health and Medical Research Council, 2006); TMD = Texture-Modified Diet; ONS = Oral Nutritional Supplement; Welch’s *t*-test and Mann–Whitney test were used to test the significance between TMDs with ONS and TMDs without ONS. *p* < 0.05 is considered significant; ^ RDI for energy and protein was calculated for individual participants and presented as mean value (SD); Carbohydrates and fat intake was calculated with their contribution to total dietary energy using their lower end of recommended intake range. Carbohydrates = 45–65% energy; Fat = 20–35% energy; Saturated fat < 8–10% energy; * Adequate intake value was used for fibre and sodium recommendations.

**Table 3:** Residents meeting the daily dietary recommendations with and without oral nutritional supplements.

% of Residents Achieved Dietary Requirement (*n*)	All TMDs *n =* 85	TMDs with ONS *n =* 46	TMDs without ONS *n =* 39	*p*-Value
Energy	35% (30)	56% (26)	10% (4)	<0.0001
Protein	72% (61)	93% (43)	46% (18)	<0.0001
Carbohydrates	39% (33)	63% (29)	10% (4)	<0.0001
Fat	76% (65)	91% (42)	59% (23)	0.0007

Note: TMD = Texture-Modified Diet; ONS = Oral Nutritional Supplement; Fisher’s Exact tests were used to test the significance of percentage differences between TMDs with ONS and TMDs without ONS. *p* < 0.05 is considered significant.

**Table 4:** Nutritional status of residents consuming texture-modified diets with and without oral nutritional supplements.

	Total (*n =* 85)	TMDs with ONS (*n =* 46)	TMDs without ONS (*n =* 39)	*p*-Value
Body weight (kg), mean (SD)	56.3 (11.8)	52.9 (9.9)	60.4 (12.8)	0.004
BMI (kg/m^2^), mean (SD)	21.8 (4.1)	20.2 (3.3)	23.5 (4.2)	0.0002
Decreased food intake % (*n*)	42% (36)	48% (22)	36% (14)	0.28
Weight loss in last 3 months % (*n*)	53% (45)	58% (27)	46% (18)	0.28
MNA-SF score, mean (SD)	8.3 (2.9)	7.4 (2.9)	9.3 (2.6)	0.005
Normal nutritional status % (*n*)	14% (*n =* 12)	7% (*n =* 3)	23% (*n =* 9)	χ^2^ (2) = 7.0, *p* = 0.03
At Risk of Malnutrition % (*n*)	48% (*n =* 41)	46% (*n =* 21)	51% (*n =* 20)	
Malnourished % (*n*)	38% (*n =* 32)	48% (*n =* 22)	26% (*n =* 10)	

Note: BMI = Body Mass Index; MNA-SF = Mini Nutritional Assessment Short-form; TMD = Texture-Modified Diet; ONS = Oral Nutritional Supplement. Nutritional status was categorised according to MNA-SF score: 12–14 = normal nutritional status; 8–11 = at risk of malnutrition; 0–7 = malnourished; Welch’s *t*-test and Mann–Whitney test were used to test the significance of weight, BMI and MNA-SF score between TMDs with ONS and TMDs without ONS. Chi-square and Fisher’s Exact tests were used to test the significance of decreased food intake, weight loss and distribution of nutritional status between groups. *p* < 0.05 is considered significant.

**Table 5:** Predictors of total energy intake and body composition (MNA score or body weight).

	Sum of Squares		Degrees of Freedom		Mean Square		F	*p*-Value
Energy Intake								
Regression	93,042,160		11		8,458,378		2.615	0.0072
Residual	236,097,298		73		3,234,210			
Total	329,139,458		84					
MNA-SF								
Regression	351.6		10		35.16		7.475	<0.0001
Residual	348.1		74		4.704			
Total	699.6		84					
	Model 1: Energy intake (kcal/day)				Model 1: MNA-SF score			
	R-square = 0.28				R-square = 0.50			
Covariates	β	SE	t	*p*-value	β	SE	t	*p*-value
Age	−28.02	27.02	1.037	0.303	−0.08	0.031	2.572	0.012
Gender ^a^	393.4	492.9	0.798	0.427	−0.43	0.592	0.722	0.472
Level of Care ^b^	−291.3	541.2	0.538	0.592	−0.44	0.650	0.678	0.500
Length of stay	3.00	6.19	0.484	0.630	0.013	0.007	1.741	0.086
Mobility level ^c^	−151.5	305.1	0.497	0.621	−0.66	0.360	1.838	0.070
Feeding assistance ^d^	124.4	265.4	0.469	0.641	−0.81	0.306	2.660	0.0100
TMD level ^e^	276.0	248.7	1.109	0.271	0.76	0.287	2.655	0.0100
Weight	52.63	33.39	1.576	0.119	−0.015	0.040	0.363	0.717
BMI	−195.6	102.1	1.915	0.059	0.32	0.118	2.681	0.009
ONS ^f^	1659	459.3	3.613	0.0006	−0.72	0.548	1.315	0.193
MNA-SF	115.8	96.39	1.201	0.234				

Note: SE = Standard Error; BMI = Body Mass Index; MNA-SF = Mini Nutritional Assessment Short-form; TMD = Texture-Modified Diet; ONS = Oral Nutritional Supplement; Multiple linear regression analysis was used: Energy intake ~ Intercept + Age + Gender + Level of care + Length of stay + Mobility level + Feeding assistance + TMD level + Weight + BMI + MNA-SF + consumption of ONS; MNA-SF ~ Intercept + Age + Male + Level of care + Length of stay + Mobility level + Feeding assistance + TMD level + Weight + BMI + consumption of ONS; ^a^ Reference category = Female; ^b^ Reference category = Hospital level; ^c^ Reference category = Mobile; ^d^ Reference category = No feeding assistance; ^e^ Reference category = Level 4—pureed diet; ^f^ Reference category = No supplements.

## Data Availability

The datasets used and/or analysed during the current study are available from the corresponding author on reasonable request.
